# Diseases Associated with Lymphatic Enlargement

**Published:** 1906-04-21

**Authors:** 


### DISEASES ASSOCIATED WITH LYMPHATIC ENLARGEMENT.

Dr. James Galloway, in a recent lecture [1] on the above subject, brought before his audience the following examples:—

1. A case of lympho-sarcoma. The patient was a boy of seventeen years, who was admitted to hospital on account of urgent dyspnœa. He presented upon examination large glandular masses in various parts of the body. The largest visible masses were in the neck, but skiagraphy demonstrated a marked invasion of the mediastinal lymphatic glands. The initial spasm of dyspnœa passed off as a result of rest in bed, but a subsequent attack led to the performance of tracheotomy. In spite of the operation, however, the lad remained slightly cyanosed, the obstruction therefore being clearly, in part at least, below the neighbourhood of the glottis. The history of the illness was to the effect that the enlargement of the glands was first observed some six months before the admission of the patient. Since that date the increase in size of the glands had been gradual but constant, while the individual glands, which were at first definitely discrete and movable, were steadily becoming more confluent. The blood-count in this case, excepting a slight degree of anæmia of chlorotic type, departed little from the normal.

2. A case of lymphadenoma. The patient was a man of middle age who first observed enlargement of the glands of his neck some eighteen months before he came under notice: at about the same time he began to be affected with a certain amount of dyspnœa. On examination he presented an enlargement of all accessible lymphatic glands, but there was no tendency for the glands to become confluent. The patient's health was not greatly impaired, while his blood-count showed no abnormality of note, excepting a mild leucocytosis.

3. A case of glandular tuberculosis simulating lymphatic leuchæmia. The patient was a young woman of twenty-one years, who some months before began to lose her health and to feel incapable of the active out-of-door life to which she had been accustomed. Examination showed that she was febrile, anæmic, and obviously ill, while the cervical lymphatic glands presented a well-marked enlargement. A blood-count showed a marked degree of anæmia, and a slight leucocytosis, but the proportion of various leucocytes departed little from the normal. The fever was continuous, and the subsequent softening and suppuration of several of the glandular masses pointed to the tuberculous nature of the infection.

4. Syphilis simulating lymphadenoma.—The case was mentioned of a young woman who was admitted to hospital as a case of lymphadenoma. The glands in the neck were enlarged, but showed no tendency towards suppuration, while very shortly after her admission many glands in many parts of the body underwent a similar enlargement. The key to the diagnosis was found when an indurated crack was noted in the centre of the lower lip. This had been the point of inoculation. Corroboration of the cause of the lesion was presented later by the appearance of a severe cutaneous syphilide.

5. A case of lymphatic leuchæmia. The patient was a woman of 55 years, who had been gradually losing health and strength for six or eight months. She presented on examination an enlargement of glands everywhere, not only in such ordinary situations as the neck, axillæ and groins, but also in the abdomen and in various parts of the body under the skin. In this case the initial opinion was that the malady was malignant disease, but an examination of the blood revealed the fact that there was a very high leucocytosis indeed, and that of the leucocytes no fewer than 93 per cent. were lymphocytes, and the great majority of these large lymphocytes.

6. By way of contrast the lecturer showed a patient who had been under observation for two and a half years. When first seen she had presented a typical picture of spleno-medullary leuchæmia, with a blood-count showing a large leucocytosis which included 34 per cent. of myelocytes. A great improvement in the condition had followed a systematic course of exposures to the Röntgen ray.

As regards treatment, the author speaks in high terms of the administration of arsenic. The drawback to this drug is its tendency to produce gastritis. For this reason he suggests the propriety of giving the agent by hypodermic injection. "A preparation which is serviceable, and which has certainly done good in cases under my own observation, is the solution of the arsenate of iron prepared for hypodermic injection by Messrs. Squire. It is made in two strengths: the milder solution contains $\frac{1}{16}$ grain of iron with $\frac{1}{180}$ grain of arsenious acid in the cubic centimetre: the stronger solution contains $\frac{1}{8}$ grain of iron with $\frac{1}{90}$ grain of arsenious acid in the same quantity of the injection. It is well in commencing treatment to use the milder of the two solutions on alternate days, proceeding to the stronger solution if it be found advisable. The solution is very little irritating, and can be borne even by sensitive individuals."

[1] Clin. Jour., March 21st, 1906.

